# Effectiveness of a Continuous Remote Temperature Monitoring Program to Reduce Foot Ulcers and Amputations: Multicenter Postmarket Registry Study

**DOI:** 10.2196/46096

**Published:** 2024-01-29

**Authors:** Chia-Ding Shih, Henk Jan Scholten, Gavin Ripp, Kirthana Srikanth, Caileigh Smith, Ran Ma, Jie Fu, Alexander M Reyzelman

**Affiliations:** 1 California School of Podiatric Medicine at Samuel Merritt University Oakland, CA United States; 2 Keck School of Medicine University of Southern California Los Angeles, CA United States; 3 Siren Care Inc San Francisco, CA United States; 4 Premier Podiatry & Orthopedics Sacramento Roseville, CA United States; 5 Samuel Merritt University San Francisco, CA United States; 6 Department of Surgery University of California San Francisco San Francisco, CA United States

**Keywords:** neuropathy, neuropathic foot ulcer, diabetes, diabetic foot ulcer, amputation, remote patient monitoring, temperature monitoring, prevention, socks

## Abstract

**Background:**

Neuropathic foot ulcers are the leading cause of nontraumatic foot amputations, particularly among patients with diabetes. Traditional methods of monitoring and managing these patients are periodic in-person clinic visits, which are passive and may be insufficient for preventing neuropathic foot ulcers and amputations. Continuous remote temperature monitoring has the potential to capture the critical period before the foot ulcers develop and to improve outcomes by providing real-time data and early interventions. For the first time, the effectiveness of such a strategy to prevent neuropathic foot ulcers and related complications among high-risk patients in a real-world commercial setting is reported.

**Objective:**

This study aims to evaluate the effectiveness of a real-world continuous remote temperature monitoring program in preventing neuropathic foot ulcers and amputations in patients with diabetes.

**Methods:**

In this retrospective analysis of a real-world continuous remote temperature monitoring program, 115 high-risk patients identified by clinical providers from 15 geographically diverse private podiatry offices were analyzed. Patients received continuous remote monitoring socks as part of the program. The enrollment was based on medical necessity as decided by their managing physician. We evaluated data from up to 2 years before enrollment and up to 3 years during the program. The primary outcome was the rate of wound development. Secondary outcomes included amputation rate, the severity of the foot ulcers, and the number of visits to an outpatient podiatry clinic after enrolling in the program.

**Results:**

We observed significantly lower rates of foot ulceration (relative risk reduction [RRR] 0.68; 95% CI 0.52-0.79; number needed to treat [NNT] 5.0; *P*<.001), less moderate to severe ulcers (RRR 0.86; 95% CI 0.70-0.93; NNT 16.2; *P*<.001), less amputations (RRR 0.83; 95% CI 0.39-0.95; NNT 41.7; *P*=.006), and less hospitalizations (RRR 0.63; 95% CI 0.33-0.80; NNT 5.7; *P*<.002). We found a decrease in outpatient podiatry office visits during the program (RRR 0.31; 95% CI 0.24-0.37; NNT 0.46; *P*<.001).

**Conclusions:**

Our findings suggested that a real-world continuous remote temperature monitoring program was an effective strategy to prevent foot ulcer development and nontraumatic foot amputation among high-risk patients.

## Introduction

### Overview

Neuropathic foot ulcers are a common complication of peripheral neuropathy. Among different etiologies leading to peripheral neuropathy, foot ulcers related to diabetic peripheral neuropathy (ie, diabetic foot ulcers [DFUs]) are the most prevalent, expensive, and deadly complications in health care [1]. Up to a third of the cost of diabetes is estimated to be related to foot care [2]. It has been reported that 10% of ulcers become infected and that 20% of infected ulcers result in an amputation [3]. While it has been reported that patients fear amputation more than death, lower extremity amputations have a close to 80% mortality rate [4,5]. DFUs also place a substantial personal burden on people and their families. Nearly half of patients report depression when they have a foot ulcer [6]. Having a foot ulcer can also cascade into other health problems when people lose their mobility, which in turn has a negative effect on the rest of their health, for example, the cardiovascular system.

Fortunately, DFUs and amputations can be prevented. Since 2007, a series of large-scale randomized control trials have shown the efficacy of temperature monitoring [7-10]. By tracking inflammation, a precursor to foot ulcers, patients and providers have an opportunity to intervene early, for example, by offloading and reducing activity. The goal is to alert people who have lost their protective sensation as early as possible of potential skin breakdown and the development of a foot ulcer. As a result of these studies, temperature monitoring is recommended in multiple clinical guidelines.

Since early 2020, a variety of remote patient monitoring (RPM) technologies have seen a rapid rise in adoption, mostly in the fields of primary care, cardiology, and pulmonology [11]. New remote temperature monitoring technologies for lower extremity care have become commercially available as well. The specific technology reported on in this study is a continuous temperature monitoring sock combined with a nursing team that monitors the data generated by the device, under the supervision of a podiatrist. Previous studies have reported on the utilization of the device and the use of the device in monitoring inflammation [12,13]. The hypothesis is that patients enrolled in the remote temperature monitoring program, designed to detect early signs of inflammation and injury, will have a statistically significant reduction in the incidence of neuropathic foot ulcers, hospitalizations, amputations, and other related complications compared with their pre-enrollment status.

### Objectives

With those new trends in mind, we wanted to study the clinical outcomes of real-world patients through a retrospective analysis before and during their use of a commercially available continuous remote temperature monitoring program.

## Methods

### Study Design

This study was from the real-world postmarket registry of an RPM program used in a commercial setting by 15 geographically diverse private podiatry practices across the state of California. This real-world study used a before-and-after study design. The design was chosen to reflect the effect of remote temperature monitoring in a real-world setting, as each patient serves as their own control group. This is an especially effective design for RPM programs and devices because device data and monitoring results are collected and transmitted in real time.

### Recruitment

The study was conducted with real-world patient data from patients who were enrolled by their provider in a remote temperature monitoring program. Given this was a real-world study, the only inclusion criterion was enrollment in the continuous remote temperature monitoring program. While the enrollment into the program was determined solely by the providers based on the patient’s medical necessity, clinical considerations included history of neuropathic foot ulcers with or without underlying peripheral arterial disease. The etiology of peripheral neuropathy includes, but is not limited to, idiopathic neuropathy, alcohol-induced neuropathy, and chemotherapy-induced neuropathy. Data of individual study participants from 2 years before enrollment were compared with data of up to 3 years during the program.

Patients from clinics that began participating in the registry study after initiating their remote monitoring program were approached if they were active within the last 12 months. We chose this cutoff because reaching out to those who left the program longer ago could be perceived as intrusive or irrelevant to their current health management.

Because this is a real-world study of an ongoing program that is offered by providers as part of their actual daily practice as opposed to a clinical trial, we did not disenroll patients. Follow-up stopped when patients no longer participated in the program; if they changed providers, changed locations, or lost or changed health insurance; could not afford copays and other out-of-pocket expenses; or stopped participating in the program for other reasons. Data from patients before they were lost to follow-up were included in the analysis of the program. The monitoring program is reimbursed by insurance and patients were responsible for any out-of-pocket expenses not covered by their insurance. Patient medical history, particularly the wound and amputation history prior to the enrollment, was reviewed based on chart review.

**Figure 1:**
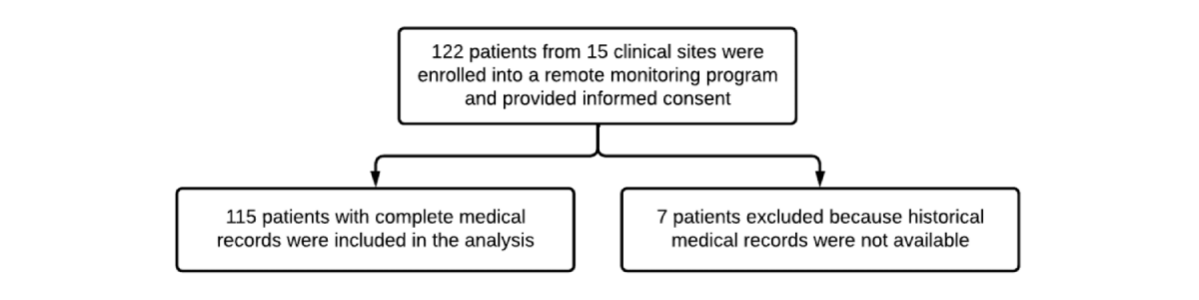
Flow diagram showing participant enrollment and dispositions.

A total of 122 patients from 15 clinical sites that were enrolled in the remote monitoring program gave informed consent, out of which 7 patients with incomplete historical medical records were excluded from the analysis population (Figure 1). Therefore, a total of 115 patients were included in this analysis. The average follow-up of this group was 14.5 (median 15.1) months, and the range was between 2 and 36 (SD 7.6) months. The reasons for early terminations are summarized in Table 1.

**Table 1:** Participant disposition.

Disposition		Participants (n=115), n (%)
Ongoing		62 (54)
**Dropoff**		53 (46)
	Lost to follow-up (unresponsive)	22 (19.1)
	Other health condition	14 (12.2)
	Product (comfort, allergy, and technical)	7 (6.1)
	Insurance related	4 (3.5)
	Lost to follow-up (patient canceled)	3 (2.6)
	Changed provider	2 (1.7)
	Deceased	1 (0.9)

### Prevention Program

**Figure 2:**
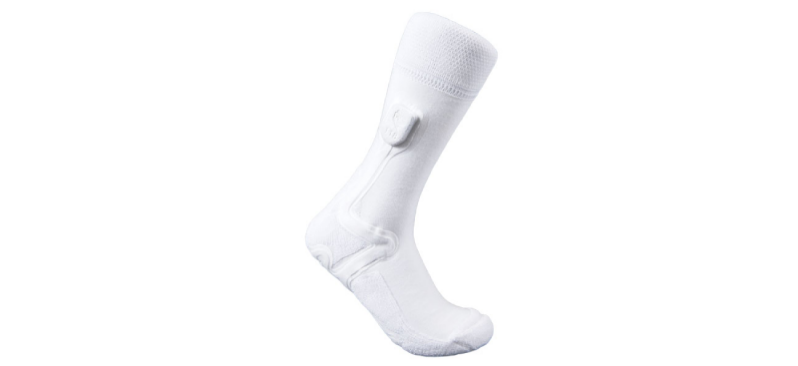
Remote temperature monitoring sock (Siren Socks, courtesy of Siren Care, Inc).

As part of the continuous temperature monitoring prevention program, patients were given continuous remote temperature monitoring socks (Figure 2; Siren Socks; Siren Care, Inc). The socks have temperature sensors embedded that collect temperature from the plantar aspect of the feet. The socks are machine washable, turn on and off automatically, and do not need to be charged. The socks are shipped directly to the patient’s home and there is no setup required. All a patient needs to do is plug in a wireless cellular data hub and put on the socks. A smartphone is not required, and the data are sent wirelessly through the data hub to the cloud.

An algorithm compares the temperature difference between the 2 feet and flags the system when a greater than 2.2 °C temperature difference is found. A 1-foot algorithm is applied for people with only 1 foot or with other amputations or deformities.

The continuous temperature monitoring prevention program also consists of a team of remote nurses who monitor the temperature data and contact a patient when a temperature difference between the feet is found. The nurses will ask the patient to reduce activity, check their feet, report symptoms, send photos, and continue wearing the socks. If the problem persists, the nurse escalates it to the patient’s managing physician—in this particular study, the podiatrist—who will decide the next steps and whether the patient needs to be seen in person at the clinic for further diagnosis and treatment as part of standard diabetic foot care.

### Measurement and Statistical Analysis

Detailed chart review and claims analysis were done and documentation, descriptions, and *International Classification of Diseases* codes in the patient’s medical chart were used to identify foot ulcers and related complications. Analysis and summary of ulcers were done by independent physicians not related to the device manufacturer.

Based on the documentation and descriptions in the medical chart, ulcers were classified for severity according to the University of Texas classification system [14].

Repeated-measures Poisson regression with an offset of the months observed in each period was used to compare the following rates before and during the program: presence of foot ulcers, ulcer severity, hospitalizations, outpatient podiatry office visits, and any lower extremity amputations. All 115 patients in the analysis population contributed before and after data for analysis; the Poisson regression model adjusts for the variable lengths of observation in the before and follow-up periods. Our choice of outcome measures aligns with those commonly reported in the literature on diabetic foot care, as well as reported in similar studies, and were determined based on their clinical relevance in the context of temperature monitoring [3,7-10,15]. The statistical analysis was performed by an independent third party not affiliated with the device manufacturer.

### Ethical Considerations

Patients from clinics participating in the registry were provided with detailed information about the study upon enrollment in the remote monitoring program and they were given the opportunity to provide informed consent for the inclusion of their data in the study. The study was reviewed and approved by WCG Clinical ethical board (WCG-IRB 1284366). All data were anonymized and deidentified.

## Results

### User Statistics

Around 91.3% (105/115) of patients had a documented diagnosis of diabetes (Table 2). Because this is a postmarket registry of a real-world private practice setting and medical necessity and enrollment were decided by the patient’s managing physician, we also observed other risk factors and forms of neuropathy, such as idiopathic neuropathy, alcohol-induced neuropathy, and chemotherapy-induced neuropathy.

**Table 2:** Patient demographics at time of enrollment (n=115).

Variables		Patient, n (%)
Age (years), mean (SD)		71.3 (9.6)
Sex (female)		44 (38.3)
Diabetes		105 (91.3)
Diabetes type I		7 (6.1)
Diabetes type II		98 (85.2)
**Race and ethnicity**		
	African American	28 (24.3)
	Asian	3 (2.6)
	Hispanic or Latino	9 (7.8)
	White	73 (63.5)
	Other	1 (0.9)
	Not documented	1 (0.9)
**Comorbidities**		
	Neuropathy	114 (99.1)
	Peripheral arterial disease	58 (50.4)
	Smoking	28 (24.3)
	Hypertension	74 (64.3)
	Kidney disease	17 (14.8)
**Foot deformity**		
	Charcot	14 (12.2)
	Hallux malleus	32 (17.8)
	Hallux valgus	11 (9.6)
	Other	24 (20.9)
History of ulcers		60 (52.2)
History of amputation		23 (20)

In our cohort, 63.5% (73/115) identified as White (58% nationally per the 2020 Census [16]), 24.3% (28/115) as African American (12% nationally), 7.8% (9/115) as Hispanic (19% nationally), 2.6% (3/115) Asian as (6% nationally), and 0.9% (1/115) were categorized as Other (6% nationally). The demographics of the at-risk population reflect the insured population in a private practice setting [16].

Around 52.2% (60/115) of patients had a previous history of ulcers, which reflects the clinical practice setting where not every patient at high risk of ulcerations has necessarily had a foot ulcer before. There are other risk factors, such as neuropathy, peripheral arterial disease, or deformities. A similar cohort was enrolled in one of the largest studies on temperature monitoring to date [8].

### Outcomes

Table 3 shows the unadjusted rates of health care use before and during the prevention program. The hospitalization rate was 63% (unadjusted rates before is 14, which is 63% lower than 39, the result during the prevention program) lower, amputations were 82% (unadjusted rates before is 3, which is 82% lower than 17, the result during the prevention program) lower, and the number of ulcers was 65% (unadjusted rates before is 33, which is 65% lower than 94, the result during the prevention program) lower.

The severity of the ulcers also decreased. Around 29% (29/99) of ulcers became infected, in line with the average of 20% [3]. During the program, 6% (2/35) of ulcers became infected.

**Table 3:** Unadjusted results before and during enrollment in program.

Outcome		Unadjusted results		
		Before		During
Total follow-up years		138.9		133.9
Average follow-up months per patient, mean (SD)		14.5 (9.5)		14 (7.6)
Average follow-up months per patient, median (range)		15.2 (2-32)		15.1 (2-36)
**Hospitalizations, n**				
	Total	39	14	
	Per patient-year	0.28	0.10	
**Outpatient office visits, n**				
	Total	1144	825	
	Per patient-year	8.2	6.2	
**Amputations, n**				
	Total	17	3	
	Per patient-year	0.12	0.02	
**Foot ulcers, n**				
	Total	94	33	
	Per patient-year	0.72	0.25	
	Per patient	0.86	0.30	
**Wound severity (before: n=99; during: n=35), n (%)**				
	1A	49 (50)	26 (74)	
	1B	15 (15)	1 (3)	
	1C	7 (7)	0 (0	
	1D	1 (1	0 (0)	
	2A	13 (13)	2 (6)	
	2B	2 (2)	1 (3)	
	2C	0 (0)	0 (0)	
	2D	1 (1)	0 (0)	
	3A	1 (1)	4 (11)	
	3B	8 (8)	0 (0)	
	3C	0 (0)	0 (0)	
	3D	2 (2)	1 (3)	
**Moderate and severe ulcers, n**				
	Total	50	9	
	Per patient-year	0.36	0.07	
	Per patient	0.43	0.08	

Table 4 shows the main outcomes and metrics of health care utilization adjusted for trends. We observed a significantly lower rate of foot ulceration (relative risk reduction [RRR] 0.68; 95% CI 0.52-0.79; number needed to treat [NNT] 5.0; *P*<.001), less moderate to severe ulcers (RRR 0.86; 95% CI 0.70-0.93; NNT 15.3; *P*<.001), and less amputations (RRR 0.83; 95% CI 0.39-0.95; NNT 41.7; *P*<.006). We also found a decrease in hospitalizations (RRR 0.63; 95% CI 0.33-0.80; NNT 5.7; *P*<.002), and a decrease in outpatient podiatry office visits during the program (RRR 0.31; 95% CI 0.24-0.37; NNT 0.46; *P*<.001).

**Table 4:** Adjusted incidence and resource use rates before and during enrollment.

Outcome	Number needed to treat	Absolute risk reduction	Relative risk reduction (95% CI)	*P* value
All foot ulcers	5.0	0.200	0.683 (0.52-0.79)	<.001
Moderate to severe ulcers	16.2	0.062	0.856 (0.70-0.93)	<.001
Outpatient podiatry visits	0.45	2.23	0.308 (0.24-0,37)	<.001
Hospitalizations	5.7	0.180	0.628 (0.33-0.80)	<.002
Amputations	41.7	0.024	0.828 (0.39-0.95)	<.006

The RRR was greater for all ulcers, hospitalization, and amputations than those observed in a previous observational study, but the absolute risk reductions were lower in this study due to lower baseline rates [15].

## Discussion

### Principal Findings

Overall, during the observation period, patients who were enrolled in the continuous temperature monitoring program at the contracted clinical sites had substantially less severe foot ulcers, fewer overall occurrences of amputations, decreased outpatient visits to their podiatrists due to early capture of potential foot wounds, and decreased rate of hospitalization. These encouraging findings suggested that the temperature monitoring socks and the prevention program were effective in preventing neuropathic foot ulcer development and recurrence as well as nontraumatic foot amputations.

### Efficacy of Continuous Remote Temperature Monitoring in the Real World

Nontraumatic amputation prevention has been a challenging task as providers often cannot capture the critical period before an ulcer has developed. The development of a neuropathic foot ulcer creates an opportunity for infection and subsequent amputations. Remote monitoring technology in foot ulcer prevention aims to help patients and providers capture signs of ulcer development. The success that was observed in this real-world study could be due to the early detection of the temperature monitoring socks followed by the foot ulcer prevention program. Our cohort exhibited a similar rate of foot ulcer prevention (absolute risk reduction 0.2, RRR 0.683, 95% CI 0.52-0.79) compared with a recent systematic review and meta-analysis focusing on temperature monitoring via thermometry (RRR 0.53, 95% CI 0.29-0.96) [17]. The program is substantially effective in preventing neuropathic foot ulcers (NNT 5.0; *P*<.001) and hospitalizations (NNT 5.7; *P*<.001), but it may be relatively less effective in preventing all types of lower extremity amputations (ie, minor and major; NNT 41.7; *P*<.006). Additionally, previously reported data suggested a relatively high rate of adherence to the program as 85% of the active patients had an average greater than 5 days per week during the program [12]. This finding may be due to different factors, including attentive nursing staff that monitored temperature changes and alerts and the ease of use of continuous temperature monitoring socks which automatically transmitted the data. From this real-world observation, the use of socks may increase compliance as opposed to other forms of remote monitoring.

### Real-World Clinical Practice and Controlled Clinical Trials

Prior studies that investigated the effectiveness of temperature monitoring were conducted in a controlled environment. Specific follow-up protocol, including outreach from clinical staff, was part of the study design. This study followed patients in real-time and real-world settings. As prior trials have established the effectiveness of temperature monitoring in the prevention of foot ulcers and amputations, our observation further validated the benefits of temperature monitoring even where patients were not specifically enrolled in a trial. This finding may be due to the enrollment of the foot ulcer prevention program in addition to the continuous temperature monitoring socks. By actively checking in with patients whose continuous temperature monitoring socks sent alerts to trained nursing staff, capturing the critical period of foot ulcer development was made possible. This study demonstrated the importance of the monitoring process as well as the continuous temperature monitoring socks.

### Real-World Clinical Scenarios and Realistic Patient Demographics

Given the presented results were based on real-world observation as opposed to a blinded randomized controlled trial, the results reflected the true use, real-world clinical scenarios, and realistic patient demographics. [18] A blinded randomized controlled trial also may not be the most ideal study design for this study as the temperature monitoring socks along with the foot ulcer prevention program would not be possible to blind either study participants or clinical providers. The observed cohort may also closely reflect podiatric practices where many high-risk patients without or with a history of foot ulcers would receive the care. This may explain a lower rate of prior foot ulcers among the cohort when compared with other controlled trials. To our knowledge, this study was the first real-world observation that investigated the effectiveness of remote temperature monitoring socks before and after their use.

### Limitations

There are a few limitations to this study. While our real-world results reflect the population demographic and clinical scenario, the observed decreased rate of recurrence and rate of amputation after patients enrolled in the continuous remote monitoring prevention program may be explained by the care from the temperature monitoring socks, the nursing team, and the involvement of the provider. Additionally, the patient population is dictated by the contracted clinical practices and patient enrollment is at the providers’ discretion. The provider’s decision to enroll patients may be limited by insurance coverage which potentially biases the results toward those with insurance coverage and adequate access to care. Nonetheless, such a real-world setting allows us to observe the real effect of the continuous remote temperature monitoring socks and the implanted care process. Another limitation is the challenge of adjusting for the disease process and other potential confounders due to the before-and-after study design. We also observed a possibly confounding factor as providers enrolled patients with other risk factors and forms of neuropathy, such as idiopathic, alcohol-induced, and chemotherapy-induced neuropathy. Given the continuous remote temperature monitoring socks are visible to patients, blinding and randomization, although effective to mitigate bias, may not be suitable in this case. Furthermore, patients opted to enroll in an insurance-covered service to prevent foot ulcers. It will be unethical to randomize patients especially when clinical providers recommend patients to enroll and subscribe for the continuous remote monitoring prevention program. Potentially, a head-to-head study in the future comparing the patients who opt out of the prevention program to those who are in the program may delineate the impacts of the program. We analyzed 115 patients from 15 sites in a single state in the United States. Although this study can benefit from a larger sample size to improve generalizability, the sample size is in line with similar studies [7-9,15]. A follow-up study with patients from multiple states is in progress to capture a larger population with more diverse demographics, health systems, geography, and cultural factors. The protocol did not allow access to medical records for the period after a patient was no longer enrolled in the monitoring program. As a result, this study provides valuable insights into the outcomes of patients during the remote monitoring program, it does not capture the outcomes after the program for those patients who discontinued the program but remained under clinical care from their provider. We will consider this for future studies or analyses.

### Conclusions

We observed substantially less ulcers, less moderate to severe ulcers, and less amputations during the foot ulcer prevention program using continuous temperature monitoring socks and a decrease in outpatient podiatry visits. Our findings suggested that a real-world continuous remote temperature monitoring program was an effective strategy to prevent neuropathic foot ulcer development and subsequent amputation among high-risk patients with diabetes. Future studies may further investigate the potential cost savings in such a strategy.

## Abbreviations

DFU: diabetic foot ulcer
NNT: number needed to treat
RPM: remote patient monitoring
RRR: relative risk reduction
